# Quality of life changes following inpatient and outpatient treatment in obsessive-compulsive disorder: a study with 12 months follow-up

**DOI:** 10.1186/1744-859X-12-4

**Published:** 2013-02-22

**Authors:** Elisabeth Hertenstein, Nicola Thiel, Nirmal Herbst, Tobias Freyer, Christoph Nissen, Anne Katrin Külz, Ulrich Voderholzer

**Affiliations:** 1 Department of Psychiatry and Psychotherapy, University Medical Center Freiburg, Hauptstraße 5, 79104, Freiburg, Germany; 2 Schön Klinik Roseneck, 83209, Prien am Chiemsee, Germany

**Keywords:** Obsessive-compulsive disorder, Quality of life, Cognitive behavioral therapy, Exposure, Inpatient, WHOQOL, Functional impairment

## Abstract

**Background:**

Quality of life (QoL) is increasingly recognized as a critical outcome parameter in mental health studies. The aim of this study was to investigate different domains of the QoL in persons with obsessive-compulsive disorder (OCD) before and after a multimodal, disorder-specific in- and outpatient treatment.

**Methods:**

Data of 73 persons with OCD treated in an inpatient setting followed by outpatient treatment were analyzed. The World Health Organization Quality of Life abbreviated (a multidimensional measure of the QoL) and the Beck Depression Inventory were administered prior to (baseline) and 12 months after the inpatient treatment (follow-up).

**Results:**

At baseline, participants reported a significantly diminished psychological, social, physical, and global QoL compared to the German general population. Environmental QoL was not impaired in the present sample. The QoL was significantly improved at follow-up, except for social QoL, but remained below norm values. The QoL improvement was predicted by improvements of depressive symptoms.

**Conclusions:**

The results indicate that persons with OCD suffer from a very low QoL. The QoL was significantly improved after 12 months of intensive state-of-the-art treatment. However, the QoL indices remained considerably lower than population norm values, indicating the need for additional research into novel treatment options for persons with OCD.

## Background

Obsessive-compulsive disorder (OCD) is a prevalent mental disorder with a lifetime prevalence of 2% to 3% [1,2]. OCD is characterized by recurrent intrusive thoughts or impulses (obsessions) and repetitive behavioral or mental acts (compulsions). The disorder frequently takes a chronic course [3]. Cognitive behavioral therapy (CBT) including exposure and response prevention (EX/RP) is the most effective treatment for persons with OCD [4,5]. To date, research into treatment efficacy has largely focused on the effects at the symptom level, with the main outcome parameters being the reduction of obsessive/compulsive or depressive symptoms. In addition, the quality of life (QoL) has increasingly been recognized as an important but rather under-investigated outcome component [6,7].

QoL has been conceptualized by the World Health Organization (WHO) as ‘a multidimensional construct describing an individual’s subjective perception of their position in life in the context of the culture and value system in which they live, and in relation to their goals, expectations, standards, and concerns’ (The WHOQOL Group, 1996).

Research on the QoL is important for a number of reasons: First, information on the domains of QoL that are particularly impaired in persons with distinct disorders might allow the therapists to specifically focus on these aspects. Furthermore, advanced knowledge about particular symptoms that have a high impact on the QoL might enable us to tailor the treatment to these symptoms [8]. Finally, QoL research might help to explain the frequently observed discrepancy between clinical symptom ratings and patients’ overall ratings of the treatment effects.

A diminished QoL in persons with OCD compared to population norms has been demonstrated by a number of studies, [7,9]. However, QoL impairments in persons with OCD have not yet been well understood with respect to different QoL domains and the association between QoL and psychiatric symptoms [10]. This aspect is further complicated by the use of different QoL measures which depict different domains of the QoL.

Improvements of the QoL in persons with OCD have been described after CBT with EX/RP in several uncontrolled pre- and post-treatment studies [6,7,9,11,12] and one randomized controlled trial with a waiting-list control group [13]. Cordioli et al. [13] observed a significant improvement of the psychological, physical, social, and environmental QoL after CBT in a group format, but not after a waiting period. The therapeutic gains were maintained at 3 months follow-up. Diefenbach et al. [11] assessed the QoL in 70 outpatients with OCD before and directly after individual CBT and reported significant improvements of work, social and family functioning with large effect sizes. Norberg et al. [7] found a substantial impairment in the QoL before treatment. One subgroup showed a substantial improvement directly after CBT with EX/RP along with a reduction in OCD symptoms. In contrast, a second subgroup also demonstrated reductions in the severity of OCD symptoms but no QoL improvement, and a third subgroup did not improve on either measure. Stewart et al. [12] analyzed data of 403 persons with severe OCD and a high rate of comorbid disorders who had not responded to previous treatments. The authors observed a significant QoL improvement directly after an intensive residential treatment with daily CBT sessions in their sample. Concerning pharmacological treatment without CBT, statistically significant but small QoL improvements have been observed after treatment with either venlafaxine or paroxetine for 12 weeks [14]. Hollander et al. [10] reported that escitalopram and paroxetine were significantly superior to placebo in improving the QoL in most domains of the SF-36. However, another randomized placebo-controlled study by Koran et al. [15] found that fluvoxamine was not superior to placebo in improving the QoL.

Interestingly, QoL improvements in persons with OCD appear to be relatively independent from the reduction of OC symptoms [14]. Some studies have indicated that the severity of comorbid depressive symptoms might be more relevant than the severity of OCD symptoms [9,16].

Until now, research into potential changes in the QoL in persons with OCD after psychotherapy has largely been limited to short-term outcomes. We found only two psychotherapy studies which provide data on the QoL at follow-up time points: Mawson et al. [17] demonstrated long-term effects (114 weeks) of a combined treatment (selective serotonin reuptake inhibitor (SSRI) and EX/RP) on a social adjustment scale, and Cordioli [13] reported that the effects of their cognitive behavioral group therapy on QoL remained stable over a period of 3 months. This situation has led several authors to recommend additional research on long-term changes of the QoL based on psychometrically sound measures [7,9,11]. Research in persons with other disorders suggests that the QoL can be improved on a long-term basis. For instance, QoL improvements over a period of 12 months have been demonstrated in persons with social phobia [18] and depression [19].

The aim of our data analysis was to investigate the QoL of persons with OCD prior to and 12 months after an intense multimodal inpatient treatment based on CBT (averaging 9 weeks) and subsequent outpatient treatment. Particularly, we addressed the following research questions: (1) Do persons with OCD suffer from a diminished QoL compared to population norms? (2) Do they exhibit an improved QoL 12 months after treatment? (3) If so, are the QoL scores comparable to population norms after treatment? (4) How are QoL changes over a 1-year period related to changes in depressive symptoms?

## Methods

### Sample

The current study included patients with a primary diagnosis of OCD according to International Classification of Diseases 10 (ICD-10) criteria who had been treated for OCD at the University Medical Center Freiburg between July 2005 and March 2010 and who had completed a measure of QoL at baseline and at follow-up. Exclusion criteria were a primary diagnosis other than OCD, a diagnosis of psychosis or organic brain disorder, and an inpatient stay shorter than 1 week. We chose to include a wide range of patients because we aimed to depict the QoL of inpatients with OCD, who typically suffer from comorbid disorders and who are often prescribed psychotropic medication.

**Figure 1:**
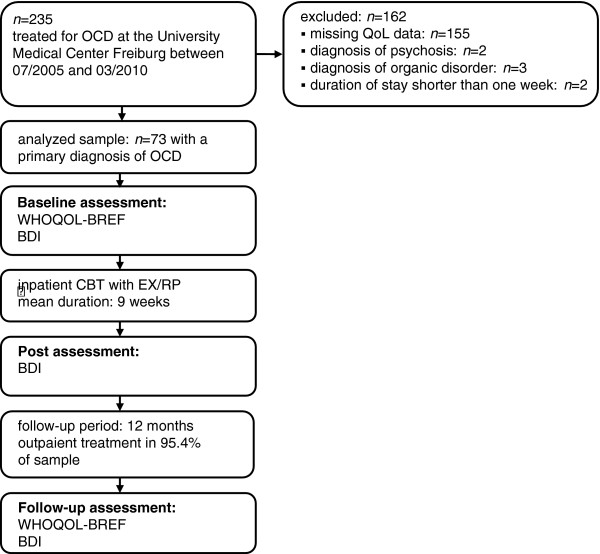
**Study design and participant flow.** BDI, Beck Depression Inventory II; *n*, total number of subjects; OCD, obsessive-compulsive disorder; QoL, quality of life; WHOQOL-BREF, World Health Organization Quality of Life abbreviated.

Of a total sample of 235 inpatients with OCD in this period, 73 fulfilled the criteria described above and were included into the present analysis. The mean age of the analyzed sample was 36 years (standard deviation 11.7 years); 39 participants (53%) were female. The participants had been suffering from OCD for an average of 15 years. Diagnoses were established by experienced clinicians based on unstructured clinical interviews. Details on the participant flow and the study design are presented in Figure 1. Demographic and clinical data of the analyzed sample and the excluded patients at baseline can be found in Tables 1 and 2.

**Table 1:** Baseline sociodemographic and clinical data I

	**Included (*n *= 73) *n *(%)**	**Excluded (*n *= 162) *n *(%)**
**Gender**		
Female	39 (53)	90 (56)
Male	34 (47)	72 (44)
**Social status**		
Unmarried	46 (63)	117 (72)
Married	21 (29)	27 (17)
Cohabiting	4 (5)	8 (5)
Other	2 (3)	10 (6)
**Occupational status**		
Employed	34 (47)	49 (30)
Student	14 (19)	29 (18)
Unemployed	9 (12)	32 (20)
Retired	8 (11)	28 (17)
Other	2 (3)	24 (15)
**School qualification**		
None	2 (3)	4 (2)
*Hauptschule*^a^	18 (25)	31 (19)
*Realschule*^a^	27 (37)	64 (40)
*Abitur*^a^	26 (35)	55 (34)
**Occupational qualification**		
None (learned)	18 (25)	41 (25)
Apprenticeship	40 (55)	83 (51)
Polytechnic	2 (3)	9 (6)
University	13 (18)	26 (16)
**Subtypes of OCD**		
Obsessions and compulsions	60 (82)	140 (86)
(F42.2)		
Mainly obsessions	7 (10)	8 (5)
(F42.0)		
Mainly compulsions	6 (8)	11 (7)
(F42.1)		
**Comorbid diagnoses**^**b**^		
Substance dependence	3 (4)	12 (7)
Mood disorder	19 (26)	53 (33)
Anxiety disorders	3 (4)	6 (4)
Eating disorders	2 (3)	2 (1)
Attention deficit hyperactivity disorder	1 (1)	4 (2)
Tourette’s syndrome	3 (4)	3 (2)
Personality disorder	2 (3)	10 (6)
Organic brain disorder	-	3 (2)
Psychosis	-	2 (1)
Bipolar disorder	-	2 (1)
Somatoform disorder	-	3 (2)

*n*, total number of subjects. ^a^German *Hauptschule* comprises 9 years of formal education, *Realschule* 10 years, and *Abitur* (A levels) 13 years. ^b^Comorbid diagnoses refer to the baseline assessment time point, not lifetime diagnoses.

**Table 2:** Baseline sociodemographic and clinical data II

	**Included (*n *= 73) mean ± SD**	**Excluded (*n *= 168) mean ± SD**	***p***
Age	35.5 ± 11.7	33.7 ± 11.1	0.26
Duration of OCD (years)	15.3 ± 11.9	15.4 ± 12.0	0.95
Age at onset of OCD	20.7 ± 11.9	19.0 ± 9.1	0.24
Inpatient stays^a^	1.9 ± 2.2	2.0 ± 2.3	0.58
BDI	18.9 ± 10.1	21.2 ± 11.0	0.15
SCL-90-R global	1.2 ± 0.7	1.2 ± 0.6	0.75
SCL-90-R OC subscale	1.9 ± 0.9	2.0 ± 0.8	0.38
GAF	41.6 ± 9.2	40.9 ± 10.2	0.60

SD, standard deviation; BDI, Beck Depression Inventory; SCL-90-R, Symptom Checklist 90 revised; GAF, Global Assessment of Functioning; *p*, level of significance for between-group comparison of included and excluded patients. ^a^The number of previous inpatient stays.

Beck Depression Inventory (BDI) scores at baseline and at discharge for the analyzed sample (*n* = 73) did not significantly differ from the scores of the *n* = 162 persons who were excluded due to missing QoL data (*p* > 0.05 for both comparisons). Baseline scores of the Symptom Checklist 90 (SCL-90-R) and the Global Assessment of Functioning (GAF) were also comparable (*p* > 0.05 for all subscales). The samples were similar concerning gender and education.

### Study design

All participants gave written informed consent for the use of their data for scientific purposes. The study design is outlined in Figure 1. Data were collected within the framework of the routine quality assurance of the hospital at three time points: baseline, discharge, and follow-up 12 months after discharge.

### Measures

#### Quality of life

The main outcome of this study was the QoL as assessed by the World Health Organization Quality of Life abbreviated (WHOQoL-BREF). The WHOQoL-BREF is a cross-culturally valid instrument with good to excellent psychometric properties [20]. The internal consistency of the German version of the instrument is strong, with Cronbach’s *α* between 0.76 and 0.88 for the different subscales [21]. A field study on the psychometric properties of the German instrument showed that subjects from the general population scored highest and psychiatric patients scored lowest on all subscales, demonstrating the construct validity of the questionnaire [21]. The WHOQoL-BREF is a short version of the WHOQoL-100 and covers four facets of the QoL, namely physical, psychological, social, and environmental QoL, in addition to a global measure. The physical QoL scale contains among others questions about physical pain, sleep, and the ability to move, whereas psychological QoL includes self-acceptance and unpleasant feelings such as sadness and anxiety. Social QoL is about relationships and social support, and environmental QoL includes questions about the housing situation, financial situation, and access to information. The questionnaire consists of 26 items which are rated on a 5-point Likert scale. For each domain of the instrument, a final score ranging from 0 (very poor QoL) to 100 (excellent QoL) can be calculated with the help of a formula provided by Angermeyer et al. [21].

#### Depression

Depressive symptoms were measured by the BDI II [22]. The BDI is a widely used self-rated instrument with good psychometric properties: BDI scores correlate strongly with the number of threshold symptoms as assessed by the Structural Clinical Interview for the Diagnostic and Statistical Manual of Mental Disorders, Fourth Edition (DSM-IV), demonstrating the questionnaire’s construct validity [22]. The same study also demonstrated a strong internal consistency (*α* = 0.92) and a high test-retest reliability (*r* = .96). BDI scores ≤ 13 can be interpreted as no or minimal depression, scores from 14 to 19 indicate mild depression, and scores ≥ 29 indicate severe depression [23].

#### Psychological distress

In order to characterize the sample, the SCL-90-R [24] was applied at baseline to assess participants’ level of psychological symptoms and psychological distress. The DSM-IV axis V GAF scale was administered as a clinician rating of global functioning, with functioning scores ranging from 1 to 100 (excellent functioning).

### Intervention

All study participants received an inpatient, multimodal, disorder-specific treatment program. The main treatment component was CBT with *in vivo* EX/RP. Treatment was carried out by experienced cognitive behavioral therapists following a structured concept [25]. The concept included extensive assessment and individual treatment planning, psychoeducative elements, *in vivo* EX/RP sessions accompanied by the therapist, as well as self-directed exposure exercises. All participants received two weekly 50-minute individual therapy sessions and participated in group therapies including a psychoeducative group, ergotherapy, music therapy, and sport therapy. Mean treatment duration was 9 weeks (SD 3.5 weeks). On admission, 43 patients (58.9% of the sample) received psychotropic medication. Thirty-seven patients (50.7%) received SSRIs, namely escitalopram (12 patients), paroxetine (12), citalopram (7), sertraline (4), fluoxetine (1), and fluvoxamine (1). Thirteen participants (17.8%) received antidepressants other than SSRIs, namely clomipramine (6), mirtazapine (3), amitriptyline (3), reboxetine (1), and venlafaxine (1). Thirteen patients received antipsychotics, namely quetiapine (5), risperidone (3), pipamperone (2), olanzapine (1), aripiprazol (1), chlorprothixen (1), flupentixol (1), and sulpirid (1). Two patients received benzodiazepines, namely lorazepam and temazepam. Two patients received opipramol. At discharge, 55 patients (75%) were on medication. Forty-seven patients took SSRIs, 18 took other antidepressants, and 13 received antipsychotics. At follow-up, thirty-eight took SSRIs, 12 other antidepressants, and 8 antipsychotics. Within the follow-up period, 95.4% of the participants underwent outpatient treatment, and 14.1% reported having been re-admitted to an inpatient setting.

### Statistics

Means and standard deviations were computed for descriptive purposes. *t* tests were calculated to compare the participants’ QoL scores to data from a norm population. Norm values are provided by Angermeyer et al. [21] for a German sample of 2,050 individuals from the general population. We conducted *t* tests for related samples to examine changes over the course of treatment. The level of significance was set at *p* < 0.05 (two-tailed). Cohen’s *d* was calculated as the ratio of the mean difference and the pooled standard deviation [26]. Effect sizes of 0.3 were considered small, 0.5 to 0.8 medium, and >0.8 large [26]. We determined clinically significant changes for individual participants according to the twofold criterion provided by Jacobson and Truax [27]. Test-retest reliability for the subscales of the WHOQoL-BREF, which is required to compute the reliable change index, was derived from Skevington et al. [20]. To investigate whether QoL changes are related to changes of depressive symptoms, we conducted a linear regression analysis. We computed difference scores (baseline minus follow-up) for the change of WHOQoL-BREF scores and the BDI. The change of WHOQoL-BREF was inserted as the dependent variable, the change of BDI scores as the predictor.

## Results

### Quality of life at baseline

At baseline, the participants’ physical, psychological, social, and global QoL was significantly diminished in comparison to population norms. Data are presented in Table 3. The impairment was most pronounced for psychological QoL (nearly two standard deviations below the norm). On the environmental subscale, the participants’ QoL did not differ from the norm value (*p* = 0.300).

**Table 3:** **QoL and depressive symptoms at baseline and 1-year follow-up for *n *= 73 patients with OCD**

	**Norm**	**OCD pre**	**OCD f-up**	**Norm vs. pre**		**Norm vs. f-up**		**Baseline vs. f-up**	
				***z***	***p***	***z***	***p***	***d***	***p***
Psych. QoL	74.0 ± 15.7	44.1 ± 19.1	59.1 ± 21.6	−1.9	**<0.001**	−0.94	**<0.001**	0.74	**<0.001**
Phys. QoL	76.9 ± 17.7	56.6 ± 16.5	70.9 ± 19.2	−1.15	**<0.001**	−0.34	**0.010**	0.80	**<0.001**
Soc. QoL	71.8 ± 18.5	54.1 ± 24.7	56.6 ± 22.7	−0.96	**<0.001**	−0.82	**<0.001**	0.11	0.316
Env. QoL	70.4 ± 14.2	68.5 ± 15.9	74.4 ± 14.6	−0.13	0.300	0.28	**0.021**	0.39	**<0.001**
Glob. QoL	67.6 ± 17.9	37.8 ± 21.5	59.2 ± 25.7	−1.66	**<0.001**	−0.47	**0.007**	0.90	**<0.001**
BDI		18.9 ± 10.1	10.3 ± 10.1					0.85	**<0.001**

QoL, quality of life; psych., psychological; phys., physical; soc., social; env., environmental; glob., global; BDI, Beck Depression Inventory; pre, baseline; f-up, 12-months-follow-up; norm, sample (*n* = 2,050) from the German general population. *z* scores were computed as follows: mean score for OCD subjects minus mean score of the norm population divided by the SD of the norm population (compare 21), *p* = level of significance (*t* test), *d* = effect sizes Cohen’s *d*. Significant results are given in bold.

### Comparison of pre- and follow-up data

#### Quality of life

**Figure 2:**
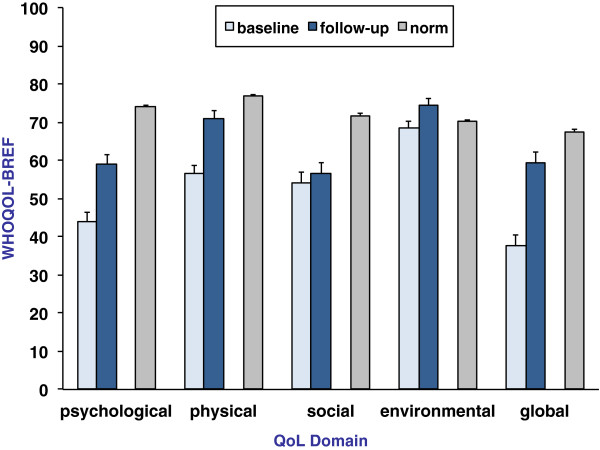
**Quality of life at baseline and 12 months follow-up.** QoL means as measured by the WHOQoL-BREF for patients with OCD at baseline and 12 months follow-up (*n* = 73) and norm values from the German general population (*n* = 2,050). Higher values indicate better QoL. Error bars represent standard errors.

Social QoL did not significantly change from baseline to follow-up. Global, physical, psychological, and environmental QoL were significantly improved. Despite these improvements, the participants’ QoL was still significantly lower than the population norms at follow-up, with the exception of the values of the environmental subscale, which were significantly higher than those of the population norm (*p* = 0.021). Data are presented in Table 3 and Figure 2. Fifteen subjects (20.3%) showed a clinically significant change in psychological QoL, 4 subjects (5.4%) in social QoL, 20 subjects (27%) in physiological QoL, and 9 subjects in environmental QoL (12.2%). Forty-nine participants (67.1%) did not show any clinically significant progress on any of the four QoL scales, and only one participant significantly improved on all four domains.

#### Depressive symptoms

BDI scores significantly declined from 18.9 (SD 10.1) at baseline to 9.6 (SD 8.8) at discharge (*p* < 0.001) and remained stable at follow-up (please refer to Table 3 for a comparison between baseline and follow-up scores). The mean BDI scores at discharge and follow-up are below the cutoff for depression.

### Relation of quality of life and depressive symptoms

BDI pre-follow-up difference scores were highly correlated with difference scores for all QoL domains (all *p* < 0.01). Linear regression analysis (Table 4) showed that the change of BDI scores was a significant predictor for the change of all QoL dimensions. The proportion of explained variance was 57.3% for global QoL, 59.2% for psychological QoL, 13% for social QoL, 19.7% for environmental QoL, and 52.6% for physical QoL.

**Table 4:** Regression analyses predicting differences in quality of life by differences in depression scores

	***R***^***2***^	***β***	***t***	***p***
Predicting global QoL	0.573	−0.757	−9.758	**<0.001**
Predicting physical QoL	0.526	−0.726	−8.885	**<0.001**
Predicting psychological QoL	0.592	−0.770	−10.088	**<0.001**
Predicting social QoL	0.130	−0.360	−3.230	**0.002**
Predicting environmental QoL	0.197	−0.443	−4.167	**<0.001**

All variables were analyzed as difference scores, baseline minus follow-up. Significant results are given in bold.

## Discussion

The results from this study indicate the following: (1) Compared to healthy subjects from the German general population, the current sample of inpatients with OCD suffered from a diminished global, physical, psychological, and social QoL with baseline scores being one to two standard deviations below norm values. Environmental QoL was not impaired. (2) QoL was significantly improved 1 year after inpatient CBT with EX/RP and subsequent outpatient treatment in all domains except social QoL. Effect sizes were medium to large for global, physical, and psychological QoL (Cohen’s *d* between 0.7 and 0.9). However, only a small subgroup of the sample showed clinically significant improvements. (3) Despite the improvements, participants’ global, physical, psychological, and social QoL remained significantly lower than population norms. (4) QoL improvements were predicted by a reduction of depressive symptoms. The amelioration of BDI scores was a good predictor of global, physical, and psychological QoL improvements with the proportion of the explained variance being greater than 50%. It is to note that the present study has relevant limitations, most prominently the lack of a severity measure of OCD and the lack of standardized diagnostic interviews. Thus, the presented results do not allow for directly investigating the impact of alterations in OCD symptoms on changes in QoL. The diagnoses were established by experienced clinicians during the inpatient stay. However, we cannot exclude that the lack of structured interviews might have biased the results.

Our results are in line with previous reports on significantly reduced QoL in persons with OCD when compared to norm values from the general population [9,10,28]. The level of impairment observed in our study corresponds well to data by Hollander et al. [10] and Albert et al. [29] who reported that baseline scores were at least one *z* score below norm values for each mental health domain of the SF-36.

With regard to distinct QoL domains, reduced QoL in the psychological domain and social functioning have been reported based on different QoL measures [7,9,10]. Skevington and McCrate [30] recently published means and standard deviations for the WHOQOL-BREF for different conditions. The score for psychological QoL of our sample was lower than the scores of persons with chronic schizophrenia, sleep disorders, diabetes mellitus, and neurodegenerative disorders. Only persons with depression reported a lower quality of life in the respective domain.

The results concerning physical QoL are controversial: Some studies found that physical QoL in persons with OCD was not impaired [31], whereas others have reported impairments [9]. One possible explanation is that impaired physical QoL in persons with OCD might reflect comorbid depressive symptoms. Moritz and colleagues [9], who found that physical QoL was impaired, reported that the criteria for major depression or dysthymia were fulfilled by 37% of their participants. In contrast, Koran et al. [31] used the same QoL measure (SF-36) and observed that physical QoL was comparable to norm values. Of note, in their sample only 2% of the participants were diagnosed with major depression and 12% with dysthymia. The differences concerning physical QoL cannot be attributed to age, as the participants of the study by Moritz et al. were younger than the sample by Koran et al. In our sample, physical QoL was reduced at baseline, which might be driven by the relatively high severity of comorbid depression (mean baseline BDI 19). More specifically, it is to note that the physical domain of the WHOQoL-BREF includes questions about the ability to manage daily routines, working ability, sleep quality, and energy for daily living. It seems plausibe that persons with mood disorders according to ICD-10 criteria who suffer from energy loss, diminished interest, and sleep disorders score low on this domain.

Environmental QoL, which is covered by the WHOQOL-BREF, but not by the SF-36, was not reduced in our sample and was slightly higher than in most other samples of persons with OCD (e.g., Cordioli [13] who found a baseline score which was more than 10 points lower than ours). Interestingly, Stengler-Wenzke and colleagues [32], who analyzed a similar German OCD sample seeking help at a University Medical Center (Leipzig), found a very similar baseline score for environmental QoL. This might be due to a selection bias. Patients with a stable socioeconomic background may be more likely to seek help in a University Medical Center in an urban area. This line of arguments fits well to the high level of education reported in our sample. Only two participants did not finish school, and 73% had at least a secondary level of education.

In line with other results, our findings indicate that the QoL of persons with OCD can be improved in the long run. In the randomized trial by Cordioli [13], the QoL was found to be improved 3 months after cognitive behavioral group therapy in comparison to baseline. This observation comprised improvements on all four domains of the WHOQOL-BREF with effect sizes within the medium range (effect sizes were calculated by the authors of the present paper based on means and standard deviations reported by Cordioli et al.).

Furthermore, we observed that the different QoL domains improved to a different extent: Improvements were most prominent for psychological and physical QoL, whereas social QoL was not significantly changed. Other studies with 12-month follow-up periods also found that social QoL did not improve in persons with depression aged 65 years or older [19] and in persons with social phobia [18]. Possibly, profound changes concerning social relationships and social support require prolonged periods of time. Insufficient social support and impaired social networks might be an etiological and maintaining factor of various mental disorders rather than a consequence of psychiatric symptoms.

Another important observation is that—despite significant improvements—the QoL of persons with OCD remained below population norms. This is in line with other results [9].

With regard to potential mediators, we found that QoL improvement was strongly linked to a reduction of depressive symptoms. Previous studies indicated that QoL and QoL improvement might be more strongly associated with depression than with OCD severity [9,16]. This implies that a careful diagnosis and, if applicable, treatment of comorbid depression is an important step in treating persons with OCD.

To the best of our knowledge, the present study is the first that repeatedly administered a psychometrically sound measure of QoL in persons with OCD over a course of 12 months. Thus, our results extend previous research by providing data on long-term changes of QoL in persons with OCD. However, a number of significant limitations need to be discussed. First, our study lacks a control group. Second, we did not control for medication. Thus, conclusions about potential causal relationships between the administration of inpatient CBT and subsequent outpatient treatment, QoL improvements, and symptomatic changes cannot be drawn. We can nevertheless draw on solid data demonstrating that OCD is a chronic disorder which does not show considerable improvement over time without treatment [33,34]. Third, the current analysis is based on quality management data from our clinic that, within the period of observation, did not routinely include measures of OCD severity. For this reason, the baseline scores of our sample were compared to data reported in other studies in order to better characterize our sample. The baseline SCL-90-R global severity indices and OC-subscale scores of our sample correspond well to the scores of *n* = 43 persons with OCD reported by Grabe et al. [35]. The baseline GAF score of 41.6 in our sample indicates severe impairment and is considerably lower than scores reported by Ramirez et al. [36] for psychiatric outpatients belonging to several diagnostic groups, including mood, anxiety, and eating disorders. Another limitation is that structured interviews have not been used. Future studies are needed to further disentangle the impact of distinct changes in psychopathology on long-term changes in the QoL. Furthermore, it remains possible that we overestimated QoL improvements, as our study suffered from a considerable amount of missing data. Patients who were less satisfied with the treatment were possibly less likely to send back the follow-up questionnaires. However, we showed that the analyzed sample did not differ from the original dataset concerning psychological distress and sociodemographic data.

## Conclusion

The present analysis shows that persons with OCD suffer from low QoL which, in the current study, improved over the course of 12 months. However, additional research on the long-term effects of CBT on QoL in the shape of a prospective, randomized, and controlled clinical trial is warranted because the present analysis has relevant limitations and does not allow for conclusive statements. Despite significant increases, four of five QoL domains remained diminished compared to healthy people. Only a minority of our patients achieved clinically significant long-term improvements of QoL. The replication of a persistently compromised QoL after state-of-the-art treatment would indicate the need for the development of novel and more effective treatments to improve the QoL in persons with OCD.

## Competing interests

CN has received speaker honoraria from Servier and has served as a scientific advisor for Novartis. The other authors report no conflicts of interest.

## Authors’ contributions

EH participated in the analysis and interpretation of the data and the drafting of the manuscript. UV, CN, and AKK participated in the conception and design of the study, the interpretation of the data, and the critical revision of the manuscript. NT, NH, and TF participated to the interpretation of the data and critical revision of the manuscript. All authors agreed to be cited as co-authors, accepting the order of authorship, and approved the final version of the manuscript.
